# Monitored anesthesia care versus general anesthesia for balloon-in-basket pulsed-field ablation of atrial fibrillation: an age-matched analysis of procedural timing and acute procedural outcomes

**DOI:** 10.1007/s10840-026-02443-2

**Published:** 2026-09-25

**Authors:** Sri Sundaram, Nicholas Palmeri, Dan Alyesh, Lukasz Cerbin, Kareena Sundaram, Ariella Rappaport, Andres Javier Dorado Montoya, Henry Zheng, William Choe, J. R. Stanton, Ahmed F. Osman, David N. Kenigsberg

**Affiliations:** 1 South Denver Cardiology Associates, AdventHealth, 1000 SouthPark Drive, Littleton, CO 80111 USA; 2 College of Arts and Sciences, Washington University in St. Louis, St. Louis, MO USA; 3 College of Literature, Science, and the Arts, University of Michigan, Ann Arbor, MI USA; 4 College of Arts and Sciences, Baylor University, Waco, TX USA; 5 Department of Anesthesiology, HCA East Florida Division, HCA Florida Westside Hospital, Plantation, FL USA; 6 Florida Heart and Vascular, Ft. Lauderdale, FL USA

**Keywords:** Atrial fibrillation, Catheter ablation, Pulsed-field ablation, Balloon-in-basket ablation, Monitored anesthesia care, General anesthesia, Procedural efficiency, Sedation

## Abstract

**Background:**

General anesthesia (GA) has been the preferred sedation strategy for atrial fibrillation (AF) catheter ablation in the United States, in part because pulsed-field ablation (PFA) systems cause skeletal muscle stimulation and discomfort that can be poorly tolerated under lighter sedation. A recently introduced balloon-in-basket (BiB) PFA system (VOLT) has been reported to produce less muscle stimulation and pain, potentially allowing Monitored Anesthesia Care (MAC) instead of GA. Whether MAC offers procedural efficiency advantages without compromising ablation efficacy in this context has not been well characterized.

**Objective:**

To compare procedural timing, ablation efficacy, and safety between MAC and GA in patients undergoing BiB PFA (VOLT) for AF, using an age-matched design.

**Methods:**

We retrospectively analyzed 39 consecutive MAC cases and a pool of 83 GA cases undergoing VOLT BiB PFA from a two-site electrophysiology registry. Each MAC patient was matched 1:1 to the nearest age available GA patient yielding 39 age-matched controls. Six different time intervals were collected: total room time, pre-procedure setup time, core procedure time, post-procedure turnover time, transseptal-to-left atrial (LA) sheath-out (dwell) time, and ablation catheter dwell time. Total ablation lesions delivered, acute procedural success (isolation of all four pulmonary veins), and procedural complications were also compared. Group comparisons were then made. In addition, EP laboratory facility cost savings and scheduling opportunity cost associated with the observed difference in total room time, using published EP/procedural-suite per-minute cost estimates were also determined.

**Results:**

Total room time (103.5 ± 20.0 vs. 126.2 ± 33.1 min; p = 0.0004), pre-procedure setup time (33.4 ± 10.0 vs. 50.3 ± 28.0 min; *p* = 0.0007), and post-procedure turnover time (14.8 ± 9.3 vs. 21.4 ± 8.3 min; p = 0.0030) were significantly shorter with MAC. Core procedure time (55.3 ± 20.5 vs. 55.3 ± 23.9 min), transseptal-to-LA-sheath-out time (34.2 ± 12.9 vs. 35.7 ± 18.5 min) did not differ significantly between groups (both *p* ≥ 0.62). Ablation catheter dwell time was modestly longer with MAC (27.7 ± 11.9 vs. 24.2 ± 15.5 min; *p* = 0.036). Total ablation lesions delivered (17.6 ± 5.1 vs. 18.8 ± 7.2, *p* = 0.68) and acute procedural success (39/39 [100%] vs. 39/39 [100%]) were similar between MAC and GA, with no procedural complications in either group. One MAC case (2.6%) was converted to general anesthesia interprocedurally for coughing attributed to airway secretions. Applying published EP/procedural-suite per-minute cost estimates ($29–$60/minute) to the observed 22.7-min reduction in total room time, MAC was associated with an estimated $658–$1,362 in facility cost savings per case, with potential opportunity cost benefit for additional case scheduling.

**Conclusion:**

In an age-matched cohort undergoing BiB PFA (VOLT) for AF, MAC was associated with shorter total room time, pre-procedure setup time, and post-procedure turnover time than GA, with no difference in ablation lesion delivery, acute success, or complications. Anesthesia related time savings with MAC appear concentrated in room logistics rather than the ablation procedure itself, with no detectable difference in acute procedural outcomes in this small cohort, supporting MAC as a reasonable alternative to GA for BiB PFA.

**Graphical abstract:**

**Figure:**
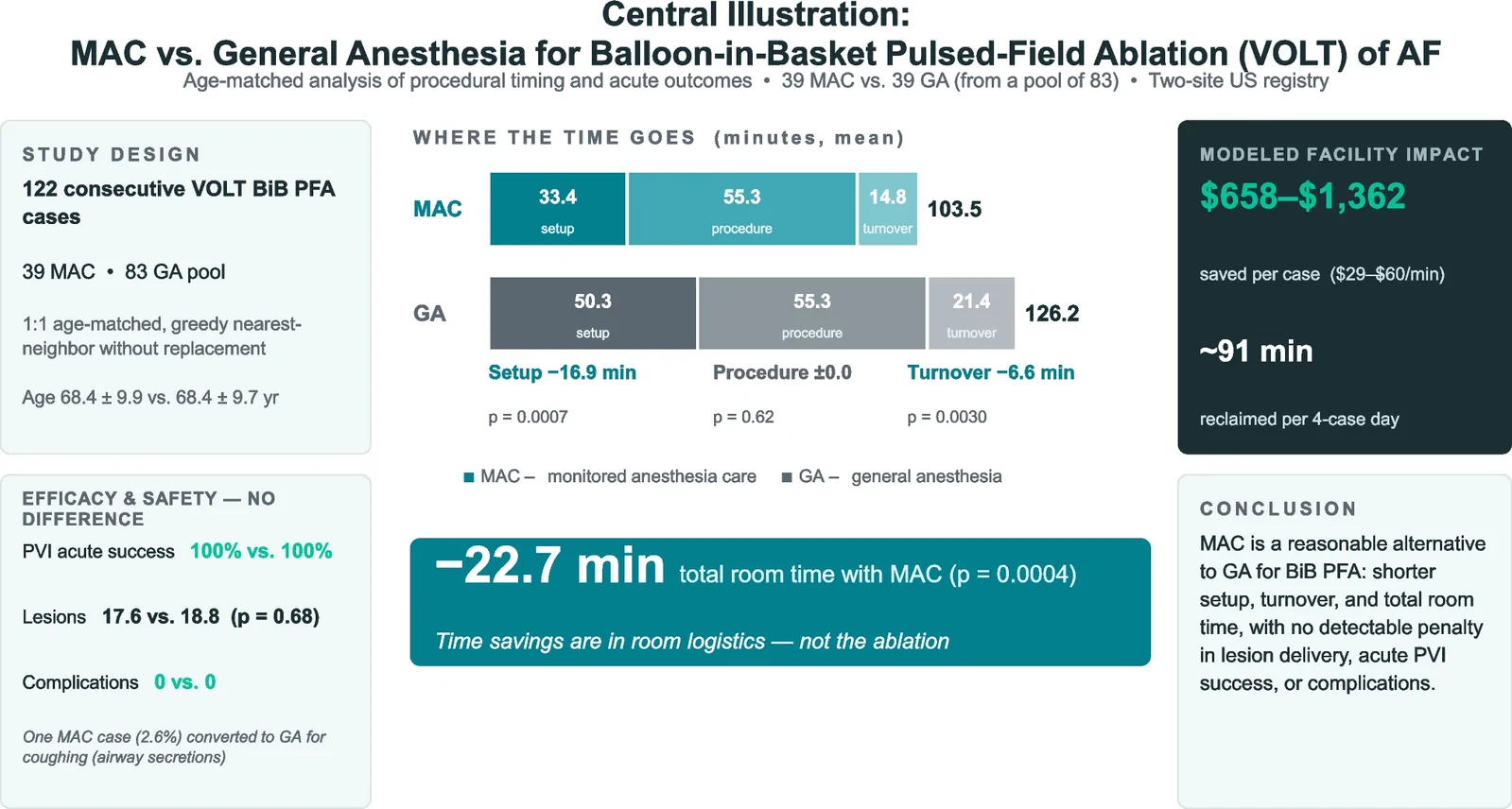

## Introduction

General anesthesia (GA) has been the predominant sedation strategy for atrial fibrillation (AF) catheter ablation in the United States, particularly for procedures using pulsed-field ablation (PFA). This practice pattern is driven in part by the biophysics of PFA energy delivery in which high voltage pulses can cause pronounced skeletal muscle and diaphragmatic stimulation, patient motion, and discomfort. In other geographies, however, Monitored Anesthesia Care (MAC) has been the standard for sedation for PFA [1–4].

A recently introduced balloon-in-basket (BiB) PFA system (VOLT, Abbott, Abbott Park, IL, USA) for pulmonary vein isolation and has been evaluated in the multicenter VOLT CE Mark study and the VOLT-AF IDE pivotal trial, with low rates of primary safety events and no observed PFA specific complications [5–7]. Emerging single center experience suggests this system may be associated with reduced pain perception and less patient movement during energy delivery compared with earlier PFA platforms. Two recent reports from Europe have described successful use of the VOLT system under moderate sedation without routine GA [2, 3]. Notably, the VOLT-AF IDE trial itself reported that conscious or deep sedation was associated with increased procedural efficiency without a difference in acute success compared with GA among 392 subjects [5]. In addition, larger real-world PFA experience points in the same direction. The EU-PORIA registry (*n* = 1233), deep sedation was associated with shorter procedure and fluoroscopy times (51 vs. 75 min), no difference in serious adverse events, and comparable one-year freedom from AF or atrial tachycardia (73.8% vs. 74.8%) [8]. If confirmed more broadly, this would represent a meaningful shift in anesthesia care for PFA for AF. Rather than being an intrinsic requirement of PFA as a modality, the need for GA may instead be a feature of specific catheter designs for appropriately selected patients. This may allow for the use of MAC, rather than GA, as a sedation strategy in the United States. In this case series, we report procedural timing and acute outcomes for MAC versus GA in BiB PFA in two US hospital based locations. This manuscript shows the timing breakdown, rather than feasibility of sedation, which has previously been established.

## Methods

### Study design and population

We conducted a retrospective cohort analysis from two hospital sites (South Denver Cardiology Associates / AdventHealth Rocky Mountain Region and Florida Heart and Vascular). All ablations in both arms were performed using either the VOLT 1 or VOLT 2 BiB PFA system (Abbott, Abbott Park, IL USA). The main difference between the two versions is that VOLT 2 catheter has improved flexibility through the shaft, more distal deflection point and shorter nose. In addition, there are shaft markers on the catheter signifying when the catheter has extended beyond the sheath. The cohort included 39 consecutive patients who underwent ablation under MAC and a pool of 83 patients who underwent ablation under GA during the same registry period. MAC in this cohort was defined as anesthesia administered without a secured airway, distinct from GA. The choice of drugs, for the MAC cohort, was left to the discretion of the anesthesia service.

### Age matching

Although the MAC and GA groups were reasonably well balanced on age prior to matching (MAC 68.4 ± 9.9 years vs. GA 68.4 ± 10.5 years; standardized mean difference [SMD] =  − 0.002), we performed 1:1 age matched control. MAC patients were identified from the cohort median using the greedy nearest neighbor without replacement method first, and each was matched to the closest age unmatched GA patient. Post-matching was balanced (MAC 68.4 ± 9.9 years vs. matched GA 68.4 ± 9.7 years; SMD < 0.001). Sex distribution and AF type (paroxysmal vs. persistent) were also well balanced both before and after matching (Table 1). All comparisons reported here use the 39 MAC vs. 39 age-matched GA cohort.

**Table 1:** Baseline characteristics before and after age matching

Characteristic	MAC (*n* = 39)	GA, full pool (*n* = 83)	*p*	GA, age-matched (*n* = 39)	*p*
Age, years (mean ± SD)	68.4 ± 9.9	68.4 ± 10.5	0.991	68.4 ± 9.7	1.000
Male sex, *n* (%)	30 (77)	59 (71)	0.647	26 (67)	0.450
Persistent AF, *n* (%)	12 (31)	40 (48)	0.106	17 (44)	0.349
BMI, kg/m2 (mean ± SD)	29.1 ± 6.0	30.9 ± 5.7	0.056	**32.6 ± 6.5**	**0.012**

Baseline characteristics pre and post age matching are shown. There was no significant difference with age, gender, persistent atrial fibrillation and BMI

### Timing variables

Six different time variables were derived from raw procedural timestamps: (1) total room time, defined as from patient time-in-room to patient time-out-of-room; (2) pre-procedure setup time, defined from time-in-room to procedure start time-out; (3) core procedure time, defined from procedure start time-out to MD scrub-out; (4) post-procedure turnover time, defined from MD scrub out to patient time-out-of-room; (5) transseptal-to-LA-sheath-out (“dwell”) time, defined as from transseptal puncture to LA sheath removal; and (6) ablation catheter dwell time, defined from ablation catheter insertion to removal. The prior metrics measure the core procedure time. The total procedure time for a physician, however, includes other variables such preparation pre-ablation and post-operative care that is not reflected in these metrics.

### Ablation efficacy and safety outcomes

To determine whether any timing differences between MAC and GA came at the expense of ablation efficacy or safety, we additionally compared: (1) total ablation lesions delivered; (2) acute procedural success, defined as isolation of all four pulmonary veins confirmed at the end of the case and (3) procedural complications. Anesthesia related safety endpoints such as conversion to GA, airway rescue maneuvers, hypotension requiring higher than usual dose of vasopressors and unexpected desaturations were also recorded.

### Statistical analysis

Group comparisons used Student's t-test when both groups were approximately normally distributed, and the Mann–Whitney U test otherwise. Baseline categorical variables were compared using chi-square tests, and binary efficacy/safety outcomes (acute success, complications) were compared using Fisher's exact test given small, expected cell counts. Analyses were performed in Python (pandas, SciPy).

### EP lab cost and opportunity cost modeling

To place the observed room time differences in an economic and operational context, we modeled EP laboratory facility cost savings and scheduling opportunity cost associated with the shorter total room time observed with MAC. We applied a per minute EP/procedural-suite cost estimate of $29 per minute, derived from our institution’s prior published analysis of EP laboratory operations, and other institutional finance data [9]. This falls within the broader published range of $22–$37 per minute for procedural-suite direct variable costs and the approximately $36–$37 per minute average reported across published operating room cost studies [10–12]. We also considered higher values such as $57.71/min as previously reported for Cath/EP labs [13]. This modeling is independent of our timing cohort and does not use patient level cost accounting or billing data from the registry. Instead, the cost analysis applies published per minute cost benchmarks to the room time difference observed in Table 2. We additionally modeled the scheduling opportunity cost of this time savings using our institution’s previously published EP lab turnover-time and scheduling data. We have previously shown that a structured workflow intervention reduced average room turnover time from 54 to 29 min and increased daily case throughput [9]. We estimated the additional case capacity that could be created within a representative EP lab day by applying the room-time savings observed here across multiple MAC cases.

**Table 2:** Procedural timing comparison, age-matched cohort (39 MAC vs. 39 GA)

Timing variable	MAC (*n* = 39)	GA, age-matched (*n* = 39)	Diff (MAC − GA), min	*p* value
Room time (in-room → out-of-room)	103.5 ± 20.0	126.2 ± 33.1	** − 22.7**	**0.0004**
Pre-procedure setup time (in-room → procedure start)	33.4 ± 10.0	50.3 ± 28.0	** − 16.9**	**0.0007**
Core procedure time (procedure start → MD scrub-out)*	55.3 ± 20.5	55.3 ± 23.9	+ 0.0	0.621
Post-procedure turnover time (scrub-out → room-out)	14.8 ± 9.3	21.4 ± 8.3	** − 6.6**	**0.0030**
Transseptal-to-LA-sheath-out (dwell) time	34.2 ± 12.9	35.7 ± 18.5	− 1.4	0.807
Ablation catheter dwell time (insert → removal)	27.7 ± 11.9	24.2 ± 15.5	** + 3.5**	**0.036**

Procedural timing was compared in the two cohorts. There was a significant difference with Total room time, Pre-procedure set up time and Post procedure turnover time. There was no differences in the core procedure time, and transseptal to LA sheath out time. (The times reported do not reflect the total time taken by a physician to compete the procedure. Scrub time and total room time underestimate the total physician time associated with a case and should not be interpreted as measures of physician workload.)

## Results

Baseline characteristics before and after age matching are shown in Table 1. The MAC and GA groups did not differ significantly in age, sex, or AF type either before or after matching, and matching further tightened age balance (SMD magnitude decreased from 0.002 to < 0.001). Timing comparisons in the age-matched cohort (39 MAC vs. 39 GA) are shown in Table 2. Total room time was significantly shorter with MAC (103.5 ± 20.0 vs. 126.2 ± 33.1 min; *p* = 0.0004). Pre-procedure setup time was also significantly shorter with MAC (33.4 ± 10.0 vs. 50.3 ± 28.0 min; *p* = 0.0007). Post-procedure turnover time was also significantly shorter with MAC (14.8 ± 9.3 vs. 21.4 ± 8.3 min; *p* = 0.0030). Core procedure time did not differ significantly between groups (55.3 ± 20.5 vs. 55.3 ± 23.9 min; *p* = 0.621). Transseptal-to-LA-sheath-out dwell time did not differ between groups (34.2 ± 12.9 vs. 35.7 ± 18.5 min, *p* = 0.807). Ablation catheter dwell time was modestly longer with MAC (27.7 ± 11.9 vs. 24.2 ± 15.5 min, *p* = 0.036).

Applying published EP/procedural-suite per-minute cost estimates to the observed 22.7-min reduction in total room time with MAC, modeled facility cost savings ranged from approximately $658 to $1,362 per case, depending on the per-minute cost assumption used Table 3. Across a representative day of four MAC cases, this corresponds to roughly 91 min of reclaimed room time and $2,633–$5,447 in modeled facility cost savings. This magnitude of time savings is comparable to what our group previously showed was sufficient to add case capacity within a fixed EP lab day through turnover-time reduction alone.

**Table 3:** Modeled EP lab facility cost savings and scheduling opportunity cost

Parameter	Assumption / Source	Modeled value
Observed reduction in total room time, MAC vs. GA	Table 2 (this study)	**22.7 min/case**
EP/procedural-suite cost per minute (published range)	Refs 9–13	$29–$60/min
Modeled facility cost savings per case	22.7 min × $29–$60/min	**$658–$1,362/case**
Modeled facility cost savings, representative 4-case MAC day	4 cases × savings/case	**$2,633–$5,447/day**
Time reclaimed, representative 4-case MAC day	4 × 22.7 min	** ~ 91 min (~ 1.5 h)**
Potential additional case capacity	Prior turnover-time reduction data (9)	≥ 1 additional short EP case, no extended lab hours

The observed difference in EP room time is 22.7 min. The estimated cost per minute from the available literature is $29-$60/min. With the time savings of 22.7 min, that equates to approximately $658 to $1362 per case. For a procedure day involving 4 cases a day, that equals 91 min less in room time or approximately $2633-$5447 per day saved. The opportunity cost of adding on an additional case with the extra 91 min is variable depending on the case

Ablation efficacy and safety outcomes did not differ between MAC and GA Table 4. Total ablation lesions delivered were similar between MAC and GA (17.6 ± 5.1 vs. 18.8 ± 7.2; *p* = 0.68). Acute procedural success, defined as isolation of all four pulmonary veins, was achieved in 100% of MAC cases and 100% of GA cases (39/39 vs. 39/39, *p* = 1.00), and no procedural complications occurred in either group. One MAC case (1/39, 2.6%) was converted to general anesthesia interprocedurally for coughing attributed to airway secretions. This was recorded as an anesthesia-related safety event rather than an ablation complication.

**Table 4:** Ablation efficacy and safety outcomes, age-matched cohort (39 MAC vs. 39 GA)

Outcome	MAC	GA, age-matched	*p*
Total ablation lesions (mean ± SD)	17.6 ± 5.1 (*n* = 39)	18.8 ± 7.2 (*n* = 39)	0.68
Acute procedural success, *n* (%)	**39/39 (100)**	**39/39 (100)**	1.00
Procedural complications, *n* (%)	0/39 (0)	0/39 (0)	1.00

There was no difference in the acute procedural outcomes of total ablation lesions and procedural success defined as pulmonary vein isolation. In addition, there were no complications in either cohort

## Discussion

In this age matched analysis, MAC was associated with significantly shorter total room time, pre-procedure setup time, and post-procedure turnover time compared with GA. Intervals specific to the ablation procedure itself, such as transseptal to Left Atrium (LA) sheath out dwell time was similar between groups, although ablation catheter dwell time was modestly longer with MAC (27.7 vs. 24.2 min; *p* = 0.036). This pattern suggests that the time advantage associated with MAC in our cohort is concentrated in anesthesia induction/emergence and room logistics (setup and turnover), rather than in the efficiency of the ablation procedure itself. This is a plausible finding as GA requires induction, airway securement, and a more involved emergence and recovery process, whereas MAC sedation can typically be initiated and reversed more quickly. Once the ablation catheter is in the LA, however, the anesthetic technique would not be expected to change catheter handling speed, lesion delivery time, or sheath management, which is consistent with what we observed.

These findings directly address the clinical question of whether MAC is a viable alternative to GA specifically for VOLT BiB PFA. GA has historically been favored for PFA in the US at least in part because of concerns about patient movement and discomfort from skeletal muscle stimulation with the currently available PFA catheters (1). Our data show that, with the BiB platform, MAC not only offers shorter room and setup times but does so without any reduction in ablation lesions delivered or acute procedural success, and without an increase in complications. This is concordant with recent single-center reports describing feasibility of conscious or moderate sedation, specifically with the VOLT BiB system [1, 2, 14], and with the larger VOLT-AF IDE and EU-PORIA experiences, in which sedation was associated with greater procedural efficiency and no difference in acute success or serious adverse events [5, 8]. Taken together, these observations support the hypothesis that the historical association between PFA and GA reflects catheter-specific biophysics rather than an intrinsic property of pulsed-field energy delivery, and that BiB systems may allow a broader range of appropriately selected patients to avoid GA without a trade-off in ablation efficacy.

Our finding of shorter total room time with MAC is directionally consistent with the pre-procedure timing pattern described by Minciună et al. and with the overall cost/time findings of Yokokawa et al., who reported longer total anesthesia care with GA, even though ablation time itself was 5 min shorter with GA in their series [15, 16]. Our data extend this by isolating the specific phase (setup, not ablation delivery) responsible for the time difference. Conversely, other work has found the opposite direction of effect. For instance, Moravec et al. found conscious sedation was associated with longer procedure and fluoroscopy times than GA, while Minciună et al. found GA improved overall efficiency specifically in the context of high-power short-duration ablation [15, 17]. This heterogeneity likely reflects differences in ablation technology (conventional vs. high-power short-duration protocols), institutional anesthesia workflows, operator experience with each technique, and case mix. A mechanistic explanation is also plausible for this difference. A recent pulmonary vein isolation series reported greater catheter swing, longer interlesion distance, and a higher rate of acute pulmonary vein reconnection with conscious sedation than with GA [18]. That mechanism is specific to catheter stability during point-by-point radiofrequency delivery and would not be expected to apply to a BiB form factor, in which the catheter is seated at the vein antrum during energy delivery. Our findings also underscore that anesthesia related time effects may not generalize uniformly across practice settings, and that phase specific timing data (as opposed to total core case time alone) may be more informative when comparing across institutions. Beyond acute endpoints, a 2025 systematic review of 23 studies comprising 12,302 patients found no difference in atrial tachyarrhythmia recurrence between sedation and GA (RR 1.15; 95% CI 0.97–1.36), as did a 1002-patient prospective multicenter registry and the Yokokawa cohort at 43 ± 17 months of follow-up [19]. These data support the absence of an efficacy penalty considerably more robustly than our acute pulmonary vein isolation comparison at 39 patients per arm.

Clinically, this granularity has practical implications for laboratory scheduling and throughput. If the time differential between MAC and GA is driven predominantly by setup and turnover rather than ablation delivery, efficiency gains from favoring MAC in appropriately selected patients would be expected to scale with the number of daily setup/turnover cycles rather than with ablation complexity. This is relevant to programs considering block scheduling, add on case capacity, or anesthesia staffing models. It also reframes the anesthesia selection discussion. The choice between MAC and GA can be made primarily on clinical grounds (airway risk, sleep apnea, expected procedure complexity, patient tolerance, need for high-frequency jet ventilation or apnea for specific ablation strategies) without an expected efficiency penalty during the ablation itself.

Our modeled EP lab cost analysis reframes the economic argument toward facility level costs and scheduling capacity. This is where the room time advantage we observed with MAC is most likely to translate into real economic benefit. Applying published EP/procedural-suite per minute cost estimates ranging from $29 per minute, derived from our own institution’s prior published EP lab cost data, to $50–$60 per minute, consistent with more recently published operating-room and procedural-suite cost benchmarks and commonly used hospital value-analysis assumptions for cath/EP labs. The 22.7-min reduction in total room time associated with MAC corresponds to an estimated $658–$1,362 in facility cost savings per case [9–12]. Because EP lab throughput ultimately governs a program’s clinical and financial capacity, we also considered the opportunity cost of this time savings in terms of scheduling. Our group has previously shown that systematically reducing EP lab turnover time, from an average of 54 min to 29 min through a structured 5-step scheduling and workflow intervention directly increased the number of cases that could be performed in a single lab day [9]. Applying the room-time savings observed here across a representative day of MAC cases suggests that shifting eligible patients from GA to MAC could reclaim enough time to accommodate at least one additional short EP case without extending lab hours. The clinical and financial value of the opportunity cost of an additional case slot, may represent the more clinically meaningful economic consequence of a MAC anesthesia strategy.

Core procedure time as described in this manuscript, does not reflect total case time. The time a physician is scrubbed in, or what is reported as "procedure time" in a case log, captures only the interval from incision to closure. It does not reflect the total physician work involved in the case. Pre-procedure work such as chart review, patient positioning, anesthesia coordination, mapping system setup, sheath and catheter preparation is not included. In addition, post-procedure work, such as hemostasis, immediate post-ablation monitoring for complications, documentation, discussion with patient and family and handoff is not included. All these additional factors fall entirely outside the core procedure time window, yet are physician directed and clinically necessary. Scrub time and total room time therefore underestimate the total physician time associated with a case and should not be interpreted as measures of physician workload.

## Limitations

This study has several limitations. First, it is a retrospective, non-randomized comparison with a small sample size. The MAC cases were also consecutive and therefore likely later in the institutional experience, so a learning-curve effect cannot be separated from the effect of anesthesia, particularly as VOLT procedure times have been reported to fall substantially with operator experience. Other variables could plausibly influence both anesthesia-type selection and procedural duration such as body mass index, obstructive sleep apnea, prior ablation, operator, and case complexity. Notably, BMI was significantly lower in the MAC group than in the age-matched GA controls (29.1 ± 6.0 vs. 32.5 ± 6.4 kg/m2, *p* = 0.013). Because lower BMI could independently shorten patient positioning, vascular access, and room turnover, this imbalance is a plausible confounder of the observed timing differences rather than evidence specific to anesthesia technique. These variables were not matched or adjusted for and could confound the observed associations. Second, the GA pool (*n* = 83) was substantially larger than the MAC cohort (*n* = 39) and our 1:1 matching approach used only 39 of the 83 available GA cases. Third, our sample size (*n* = 39 per group), still limits precision, particularly for the nonparametric effect size estimates and the lesion-count and acute-success comparisons. Finally, this analysis reflects the practice patterns and case mix of two sites and may not generalize to institutions using different ablation technologies, anesthesia protocols, or staffing models. With regards to limitations with the EP lab cost model, it relies on published per minute cost estimates, including one derived from our own institution’s prior published data, rather than actual facility cost accounting or billing data from this registry. Our model applies a single representative case-volume assumption (four MAC cases per day) to estimate scheduling opportunity cost, and does not account for staffing, equipment, payer mix, or case-mix variability across institutions. It should be interpreted as an illustrative estimate rather than a formal cost effectiveness or capacity analysis of our specific programs. The modeled savings also do not account for offsetting differences in anesthesia professional fees and drug costs. Consistent with this, Yokokawa et al. reported only an approximately 7% increase in total charges with GA, an order of magnitude smaller than the difference implied by our model [16].

## Conclusion

In an age-matched, retrospective cohort of patients undergoing VOLT BiB PFA for AF, MAC was associated with significantly shorter total room time, pre-procedure setup time, and post-procedure turnover time compared with GA. There was no difference in ablation lesions delivered, acute procedural success, or complications. These findings suggest that anesthesia related time savings with MAC are attributable to room logistics rather than ablation efficiency, with no detectable difference in acute procedural outcomes in this small cohort. This supports MAC as a reasonable anesthesia strategy for appropriately selected patients undergoing BiB PFA. This also has potential implications for laboratory throughput and anesthesia selection strategy. Larger, prospective, multi-site studies with adjustment for additional confounders are warranted to confirm these findings and further define the role of MAC with this ablation technology.

## Data Availability

No datasets were generated or analysed during the current study.
